# Study on the collaborative protective mechanism of *Scutellariae Radix* and *Paeoniae Radix Alba* against diabetic cardiomyopathy through the gut-heart axis

**DOI:** 10.3389/fmicb.2025.1500935

**Published:** 2025-04-08

**Authors:** Cheng Fang, Xiaomin Xu, Fang Lu, Shumin Liu

**Affiliations:** Heilongjiang University of Chinese Medicine, Harbin, China

**Keywords:** *Scutellariae Radix* and *Paeoniae Radix Alba*, intestinal bacteria, intestinal mucosal barrier, serum metabolites, diabetic cardiomyopathy

## Abstract

The modification of gut microbiota has been linked to diabetic cardiomyopathy, yet the precise mechanisms through which gut microbes impact cardiac injury remain unclear. Our study concentrated on the gut microorganisms, the intestinal mucosal barrier, and the metabolic pathways involving glucose and lipids in mice afflicted with diabetic cardiomyopathy, while also investigating the cardioprotective properties of *Scutellariae Radix* and *Paeoniae Radix Alba*. Using a db (Leptin receptor gene-deficient mouse) mouse model of diabetic cardiomyopathy, we observed that these mice exhibited a decline in the diversity of intestinal microbes, alterations in the abundance of diabetes-related microorganisms, a decrease in *Firmicutes*, an increase in *Helicobacter*, and an overall rise in intestinal microbial populations. We pinpointed the inflammatory response and the compromised permeability of the intestinal lining as key contributors to the decline of the intestinal mucosal barrier, subsequently leading to cardiac injury. Administering *Scutellariae Radix* and *Paeoniae Radix Alba* was shown to restore the equilibrium of the intestinal microbiota, modify metabolic pathways involving glycerophospholipids, arachidonic acid, and additional metabolites within the myocardial tissue through bile acid, taurine, and associated metabolic processes, resulting in lessened cardiac dysfunction, hypertrophy, and fibrosis in the diabetic cardiomyopathy mice. In conclusion, our findings indicate that the intestinal microbiota, intestinal mucosal barrier, and glycolipid metabolism are disrupted in mice with diabetic cardiomyopathy; however, *Scutellariae Radix* and *Paeoniae Radix Alba* may effectively reverse these alterations. These results offer valuable insights for creating therapeutic strategies aimed at mitigating cardiac damage linked to diabetes by focusing on the gut microbiota and glucose and lipid metabolism.

## 1 Introduction

Diabetes has emerged as a significant public health challenge globally. As reported in the ninth edition of the International Diabetes Federation’s statistics from 2017, there are 425 million individuals living with diabetes around the world. Alarmingly, projections indicate that this figure may surpass 700 million by the year 2045 (Sun et al., 2022). One significant complication arising from diabetes is diabetic cardiomyopathy. This condition, known as diabetic cardiomyopathy (DCM), encompasses myocardial metabolic disturbances, issues with cardiac microvasculature, and fibrosis of the myocardium, leading to left ventricular hypertrophy as well as systolic and/or diastolic dysfunction. Recent research indicates that DCM contributes to heart failure in certain patients (Wang L. et al., 2017). Managing blood glucose levels is thought to delay the advancement of DCM during the initial phases of heart dysfunction, and it is crucial to lower hyperglycemia to prevent DCM and mitigate its effects (Atkinson et al., 2014). As heart failure research has progressed, it has become clear that beta blockers play a crucial role in managing this condition. They may not only hinder but also potentially reverse the remodeling of the heart, thereby improving left ventricular function and reducing mortality rates (Thaiss et al., 2018). Nevertheless, most of these drugs come with various drawbacks, such as significant toxicity and side effects, multiple adverse reactions, a focused target, minimal efficacy, and potential liver harm. In contrast, the compound prescriptions in traditional Chinese medicine are distinguished by their multi-component, multi-target, and multi-link characteristics, offering holistic regulation and intervention. This approach presents a novel perspective for investigating and preventing this condition. Consequently, we examined the protective mechanisms in diabetic cardiomyopathy.

Huangqin Decoction is a time-honored formula in traditional Chinese medicine, originally documented in the esteemed text “Treatise on Febrile Diseases,” authored by Zhang Zhongjing during the Eastern Han Dynasty. This medicinal formula has a rich history spanning nearly 1,800 years, reflecting its enduring relevance in the field of herbal medicine. It is commonly employed in clinical settings to address various intestinal disorders, particularly ulcerative colitis, highlighting its significance in gastrointestinal health management within traditional therapeutic practices (Feng et al., 2020; Li et al., 2024; Tang et al., 2024). Huangqin decoction has been shown to have a good therapeutic effect on complex gastrointestinal symptoms (Li et al., 2024), and its mechanism is thought to be related to its regulation of intestinal flora and inhibition of intestinal inflammation (Li et al., 2022). Research indicates that Huangqin decoction has the ability to regulate the composition, diversity, and abundance of gut microbiota, mitigate significant damage inflicted by inflammation on the intestinal barrier, and reduce the infiltration of inflammatory cells within the intestines (Li et al., 2022). Simultaneously, various elements of traditional Chinese medicine exhibit intricate effects on gut microbiota. Huangqin Decoction has undergone extensive investigation regarding its efficacy in managing diabetes and related complications. Reports indicate that Huangqin Decoction can be utilized independently for diabetes treatment, while other studies highlight its application alongside additional medications to address diabetes and its complications. This research primarily focuses on metabolomics, gut microbiota, and other related areas (Xu et al., 2022). *Scutellariae Radix* and *Paeoniae Radix Alba* are the most important two traditional Chinese medicines in Huangqin Decoction. Consequently, we aim to integrate intestinal microbiota and metabolomics techniques to explore how *Scutellariae Radix* and *Paeoniae Radix Alba* influences its metabolites via the activity of intestinal flora, and to assess its protective role on the intestinal mucosal barrier. This research seeks to establish a theoretical foundation for disease prevention and the exploration and development of novel pharmaceuticals.

Larsen et al. (2010) confirmed that diabetes patients’ intestinal flora differs significantly from that of normal individuals. At the molecular level, a study of diabetes patients and non-diabetic persons revealed that differences in intestinal microbial composition and diversity of these species were thought to be potentially associated to T2DM. The amount of *Bifidobacterium*, *Clostridium*, and *Firmicutes* in diabetes patients’ intestinal flora is much lower than in normal persons (Karlsson et al., 2013; Sato et al., 2014). While *Bacteroides* and *Proteobacteria* numbers increased significantly, and the ratios of *Bacteroidetes/Firmutes* and *Firmicutes/Clostridium* were also positively correlated with blood glucose levels, although these ratios did not seem to be related to body weight (Qiu et al., 2019). Nonetheless, a solitary investigation into intestinal microecology does not comprehensively elucidate the pathogenesis of diseases. Consequently, this experiment aims to employ the latest generation Illumina MiSeq platform’s 16S rRNA technology alongside serum metabolomics analysis. The objective is to explore the mechanisms by which *Scutellariae Radix* and *Paeoniae Radix Alba* treats diabetic intestinal mucosal damage through a multi-tiered integration of the relationships between intestinal flora and metabolites, ultimately offering novel diagnostic and therapeutic targets for clinical translational research.

Metabolomics involves both the qualitative and quantitative examination of small molecular metabolites found in biological samples. This field explores the connections between metabolites and the goals of research, playing a crucial role in identifying biomarkers for DCM. Biomarkers serve a vital function in the clinical diagnosis of various metabolic disorders, such as DCM, and are considered effective instruments for comprehensive research (Wang et al., 2015; Guo et al., 2020). The development of network pharmacology builds on the growing understanding of protein and molecular interactions, and it is extremely helpful in understanding the pathogenesis of TCM syndrome and the treatment mechanism of TCM (Petrie et al., 2018; Sonnweber et al., 2018).

Thus, the integration of network pharmacology and metabolomics offers a valuable approach to elucidate the metabolic mechanisms of DCM in addressing diabetes and its associated complications.

## 2 Experimental materials and methods

### 2.1 Experimental materials

The raw botanical materials derived from *Scutellariae Radix* and *Paeoniae Radix Alba* (SP) were acquired from Hebei Quantai Pharmaceutical Co., Ltd. The *Scutellariae Radix* (batch number: 1907002) refers to the dried root of *Scutellaria baicalensis Georgi*, whereas *Paeoniae Radix Alba* (batch number: 2001002) is the dry root from the *Ranunculaceae* family, specifically *Paeonia lactiflora* (*Paeonia lactiflora* Pall.). The following products were utilized in the research: DNeasy PowerSoil Kit (QIAGEN, The Netherlands); Quant-iT PicoGreen dsDNA Assay Kit (Invitrogen, USA); Agcout AMPure Beads (Beckman Coulter, USA); PicoGreen dsDNA Kit (Invitrogen, USA); DAB Color Reagent (Zhongshan, Beijing); CD45 antibody (Santa, USA); and PV9004 secondary antibody (Zhongshan, Beijing).

Quantifluor-ST fluorometer (Promega); high-throughput sequencer (Illumina, USA); medical freezer for low temperatures (Haier Co., Ltd.); gel imaging system gdsAUTO520 (BG Corp., USA); electrophoresis instrument DYY-6C (ABI Company, USA); paraffin embedding device (Leica EG1150H); microtome for paraffin (Leica RM 2125 RTS); another paraffin microtome (Leica HI1220); fluorescence microscopy equipment (OLYMPUS DP80).

Weigh the SP components and combine them in a ratio of 3:2. Then, introduce water in an amount equal to 10 times the overall weight of the mixture. Allow it to soak for 30 min and subsequently simmer for 1 h. After straining the filtrate, add water in an amount that is 8 times that of the remaining residue and continue to simmer for another hour. Filter this second filtrate, then merge it with the first, and concentrate the combined solution to a density of 0.75 g/mL. A sample of the concentrated solution should be taken and diluted with water at a ratio of 1:2. Ultimately, the concentration of SP at the high dose is 0.75 g/mL, while the low dose concentration is 0.25 g/mL.

### 2.2 Experimental animals

A total of forty male db/db mice and eight db/+ mice were procured from Ermaite Technology Co., Ltd., with the animal certificate number 202009670. These animals were housed in a single cage that permitted free access to food and water, ensuring they were well-nourished and hydrated. They were maintained in a clean environment that was specific pathogen free, which is crucial for minimizing the risk of infections that could negatively impact the study outcomes. The temperature within the housing facility was carefully regulated, maintained between 20 and 26 degrees Celsius. Additionally, the humidity levels were kept within a range of 40 to 70 percent, which contributes to the overall health and comfort of the animals. Lighting in the facility followed a 12-h light and 12-h dark cycle, a standard practice that helps simulate natural day-night conditions. The cages and bedding were routinely changed on a weekly basis to ensure cleanliness and to promote a healthy living environment for the mice. All procedures involving animal experimentation were carried out in compliance with the applicable guidelines established by the Laboratory Animal Ethics Committee of Heilongjiang University of Traditional Chinese Medicine. This adherence to ethical standards is essential for ensuring the welfare of the animals involved in research. The specific ethics approval number granted for this research project is 2,020,083,001, reflecting that the study has met the necessary ethical requirements prior to the commencement of any experimental activities.

The forty male db/db transgenic mice were assigned to four distinct groups: the model group, a high-dose group receiving SP at 5.625 g/kg, a low-dose group receiving the same treatment at 1.825 g/kg, and a positive control group treated with Metformin at 250 mg/kg. The db/m mice served as the control group. Following H&E (hematoxylin–eosin staining) and immunohistochemical analysis, the treatment group that exhibited the most significant effects in earlier studies was chosen for further mechanistic investigation.

### 2.3 Sample collection and processing

Mice were intraperitoneally anesthetized with pentobarbital sodium 24 h following the final treatment, and blood was collected from the ocular region. The blood samples were allowed to stand for a period of 60 min at 4°C, after which they were centrifuged (3,500 rpm for 15 min at 4°C) to separate the serum, which was then stored at −80°C for later analysis. Upon thawing at room temperature, three volumes of ice-cold methanol were added, the mixture was vortexed for 3 min, and then centrifuged twice (12,000 rpm for 15 min at 4°C). The resulting supernatant was stored at −80°C for UHPLC/MS evaluation. Following the sacrifice of the mice, cecal contents were retrieved, placed in sterile tubes, sealed promptly, frozen quickly in liquid nitrogen, and subsequently kept at −80°C for analysis of intestinal flora diversity and for immunohistochemistry detection. A segment of the intestinal tract was removed, and the intestinal contents were expelled using normal saline, then fixed in formalin solution for future H&E staining.

### 2.4 Echocardiography

Echocardiographic evaluation in anesthetized mice were performed using the Vevo 2,100 imaging system equipped with a 20 MHz ultrasound transducer. Left ventricular functional indexes were measured, and ejection fraction (EF) and fractional shortening (FS) were calculated. All the measurements were the average of three consecutive cardiac cycles and performed by the same operator.

### 2.5 H&E staining

Cecal tissues from mice were subjected to waxing and dehydration processes. The film was cut and positioned on a cover glass to allow it to dry. Prior to staining, the paraffin from the section must be dissolved using xylene, followed by gradient alcohol colorization from high to low concentration, and then immersed in distilled water. Slices that have absorbed distilled water was placed in a hematoxylin solution for several minutes to achieve coloration. The color separation occured in acidic and ammoniacal water for a few seconds; subsequently, after an hour of washing with running water, distilled water was added briefly. Dehydrate the slices in 70 and 90% alcohol for 10 min each. Finally, for 2 to 3 min, immerse the slices in the eosin staining alcohol solution.

### 2.6 Immunohistochemistry

Alterations in CD45 expression observed in the cecal tissue of diabetic mice were linked to damage in the intestinal mucosa. The cecal tissue was prepared by embedding in paraffin wax and slicing into sections. These sections were sealed in an oven set at 78°C overnight. The following day, the samples were immersed in xylene for 10 min and subsequently dehydrated using a graded alcohol series. Residual xylene was eliminated by treating the slices for 5–10 min, followed by two washes with distilled water (3–5 min per wash). The tissue was blocked with 3% hydrogen peroxide for 10 min and rinsed twice with distilled water (3–5 min per rinse). For antigen retrieval, tissue repair was performed using citric acid buffer: samples were placed in boiling water, heating was discontinued upon steam generation, and the air valve was opened after 2–3 min. The lid was removed after 7 min, and tissues were extracted and cooled at room temperature for approximately 50 min. Following cooling, samples were washed three times with PBS (5 min per wash). Primary antibody (100 μL) was applied to the preparations, which were incubated overnight at 4°C. The next day, unbound antibody was removed by three PBS washes (5 min each). Secondary antibody (100 μL) was then applied for 5-min incubations. Color development was initiated with DAB for 5–10 min, and the reaction was terminated upon microscopic confirmation of optimal staining. After rinsing with water for 3–5 min, counterstaining was performed using hematoxylin for 1 min. Excess stain was removed by a 10–15-min water rinse, with staining intensity microscopically assessed and repeated if unsatisfactory. Finally, dehydration was achieved through sequential alcohol washes (2–3 min per step), residual alcohol was cleared, and sections were sealed with neutral gum at 87°C for 2–3 min.

### 2.7 16S rRNA sequencing analysis

The sequencing platform utilized was the Illumina MiSeq. Following the implementation of double-ended data connections and chimera removal, the library information 2,100 was evaluated using a high-sensitivity quantitative approach with the library kit. To construct the library for the sample kit, TruSeq Nano DNA LT was employed; the effective sequence corresponding to the 16S rRNA V4 region was sequenced with Miseq’s 2,300 bp double-ended method through fluorescent quantitative analysis. The outcomes of the sequencing were represented and analyzed employing QIIME, R, and Mothur software.

### 2.8 Serum metabolomics

#### 2.8.1 UPLC-MS analysis conditions

The WATERS ACQUITY™ UPLC BEH C18 column (2.1 mm × 50 mm, 1.7 μm) utilized a mobile phase composed of a 0.05% formic acid acetonitrile solution (component A) and a 0.05% formic acid aqueous solution (component B). The elution gradient was set as follows: from 0 to 5 min at 1 to 40% A, from 4 to 9 min at 40 to 70% A, and from 9 to 14 min at 70 to 100% A. An injection volume of 2 μL was employed, with a flow rate maintained at 0.4 mL/min, and the temperature of the column was kept at 40°C. Detection was accomplished under mass spectrometry conditions utilizing an electrospray ionization (ESI) source operating in both positive and negative ion modes, while the Lock Mass method operated in full scan mode. The mass scanning range spanned m/z 100 to 1,500, with a spray frequency of 10 s. Leu enkephalin mass calibration was executed through Waters’ online system, with the positive ion recorded at m/z + 556.2771 [M + H] + and the negative ion at m/z 556.2615 [M-H]-. Additional mass spectrum parameters are detailed in Table 1.

**Table 1:** Information on potential biomarkers in mouse serum under positive and negative ion modes.

NO.	Rt-m/z	HMDB	KEGG	Formula	Ions mode	Description
1^B^	4.81_495.3367n	HMDB10382	C04230	C_24_H_50_NO_7_P	Pos	LysoPC(16:0)
2^B^	7.22_782.5916 m/z	HMDB07954	C00157	C_45_H_86_NO_8_P	Pos	PC(15:0/22:2(13Z,16Z))
3^B^	5.34_521.3563n	HMDB02815	C04230	C_26_H_52_NO_7_P	Pos	LysoPC(18:1(9Z))
4	7.21_825.6087n	HMDB09113	C00350	C_47_H_88_NO_8_P	Pos	PE(18:2(9Z,12Z)/24:1(15Z))
5^B^	6.16_523.3622n	HMDB10384	C04230	C_26_H_54_NO_7_P	Pos	LysoPC(18:0)
6	4.54_519.3408n	HMDB10386	C04230	C_26_H_50_NO_7_P	Pos	LysoPC(18:2(9Z,12Z))
7^B^	1.30_102.0473n	HMDB00060	C00164	C_4_H_6_O_3_	Pos	Acetoacetic acid
8^B^	8.16_785.6206n	HMDB09512	C00350	C_45_H_88_NO_7_P	Pos	PE(22:0/P-18:1(11Z))
9	3.66_414.2046n	HMDB14838	C12512	C_24_H_30_O_6_	Pos	Eplerenone
10	7.22_832.5962 m/z	HMDB07988	C00157	C_46_H_84_NO_8_P	Pos	PC(16:0/22:4(7Z,10Z,13Z,16Z))
11^B^	8.33_810.6229 m/z	HMDB09511	C00350	C_45_H_90_NO_7_P	Pos	PE(22:0/P-18:0)
12	7.21_834.6218 m/z	HMDB09776	C00350	C_47_H_90_NO_7_P	Pos	PE(24:1(15Z)/P-18:1(11Z))
13^B^	8.16_813.6889 m/z	HMDB12107	C00550	C_47_H_93_N_2_O_6_P	Pos	SM(d18:1/24:1(15Z))
14	8.16_834.6164 m/z	HMDB08046	C00157	C_46_H_86_NO_8_P	Pos	PC(18:0/20:3(5Z,8Z,11Z))
15	5.06_518.3269 m/z	HMDB10387	C04230	C_26_H_48_NO_7_P	Pos	LysoPC(18:3(6Z,9Z,12Z))
16^B^	4.82_184.0755 m/z	HMDB00510	C00956	C_6_H_11_NO_4_	Pos	Aminoadipic acid
17^B^	3.08_274.2778 m/z	HMDB00220	C00249	C_16_H_32_O_2_	Pos	Palmitic acid
18^B^	2.07_192.0640 m/z	HMDB00001	C01152	C_7_H_11_N_3_O_2_	Pos	1-Methylhistidine
19^B^	6.32_550.3924 m/z	HMDB10391	C04230	C_28_H_56_NO_7_P	Pos	LysoPC(20:1(11Z))
20	3.00_322.1541n	HMDB15204	C12755	C_20_H_22_N_2_S	Neg	Mequitazine
21^B^	0.69_215.0374 m/z	HMDB00318	C05576	C_8_H_10_O_4_	Neg	3,4-Dihydroxyphenylglycol
22^B^	7.48_596.3939 m/z	HMDB10390	C04230	C_28_H_58_NO_7_P	Neg	LysoPC(20:0)
23^B^	4.97_590.3565 m/z	HMDB10393	C04230	C_28_H_52_NO_7_P	Neg	LysoPC(20:3(5Z,8Z,11Z))
24	4.77_614.3482 m/z	HMDB10402	C04230	C_30_H_52_NO_7_P	Neg	LysoPC(22:5(4Z,7Z,10Z,13Z,16Z))
25	3.97_586.3164 m/z	HMDB10397	C04230	C_28_H_48_NO_7_P	Neg	LysoPC(20:5(5Z,8Z,11Z,14Z,17Z))
26^B^	2.52_514.2894 m/z	HMDB00036	C05122	C_26_H^45^NO_7_S	Neg	Taurocholic acid
27^B^	2.16_321.0432 m/z	HMDB01227	C00364	C_10_H_15_N_2_O_8_P	neg	5-Thymidylic acid
28^B^	1.67_212.0019 m/z	HMDB00232	C03722	C_7_H_5_NO_4_	Neg	Quinolinic acid
29^B^	1.04_269.0875 m/z	HMDB00034	C00147	C_5_H_5_N_5_	Neg	Adenine
30^B^	0.69_395.0969 m/z	HMDB00291	C05584	C_9_H_10_O_5_	Neg	Vanillylmandelic acid
31^B^	0.69_217.0329 m/z	HMDB00965	C00519	C_2_H_7_NO_2_S	Neg	Hypotaurine

Rt-m/z refers to the retention time of mass-to-charge ratio; Formula refers to the marker molecular formula; Description refers to the marker name; Ions mode refers to the ionicity of the charge; B refers to the metabolites with significant differences.

#### 2.8.2 Metabolomic analysis of potential markers

The mass spectrum peak created from the ions obtained is illustrated through the total ion flow chromatogram produced by UPLC-TOF-MS. Using Qi and MarkerLynx XS software, the molecular weight of the metabolites can be determined, and the acquired data is subsequently processed in EZinfo 2.0 software after eliminating external interference components. Principal components analysis (PCA) is employed to analyze data from serum samples of mice, with a scores plot used to describe the level of dispersion among groups. The study then evaluates the similarity of the trajectories of serum samples across different groups to assess any clustering effects. Potential biomarkers are identified through a combination of s-plot and projection screening methods, focusing on ion variable importance (VIP) values greater than 1. Furthermore, the biological significance of these biomarkers is evaluated by integrating information from the human metabolomics database (HMDB) and the Kyoto Encyclopedia of Genes and Genomes (KEGG), along with a review of related literature.

### 2.9 Statistical methods

The data obtained from every group were evaluated through the SPSS 20.0 statistical software, and the findings are expressed as x ± s. One-way ANOVA was utilized to analyze the data, and the least significant difference (LSD) test was conducted for group comparisons; a statistically significant difference was noted with *p* < 0.05.

## 3 Results

### 3.1 The effect of SP on glucose metabolism and protect cardiac function in db mice

**Figure 1:**
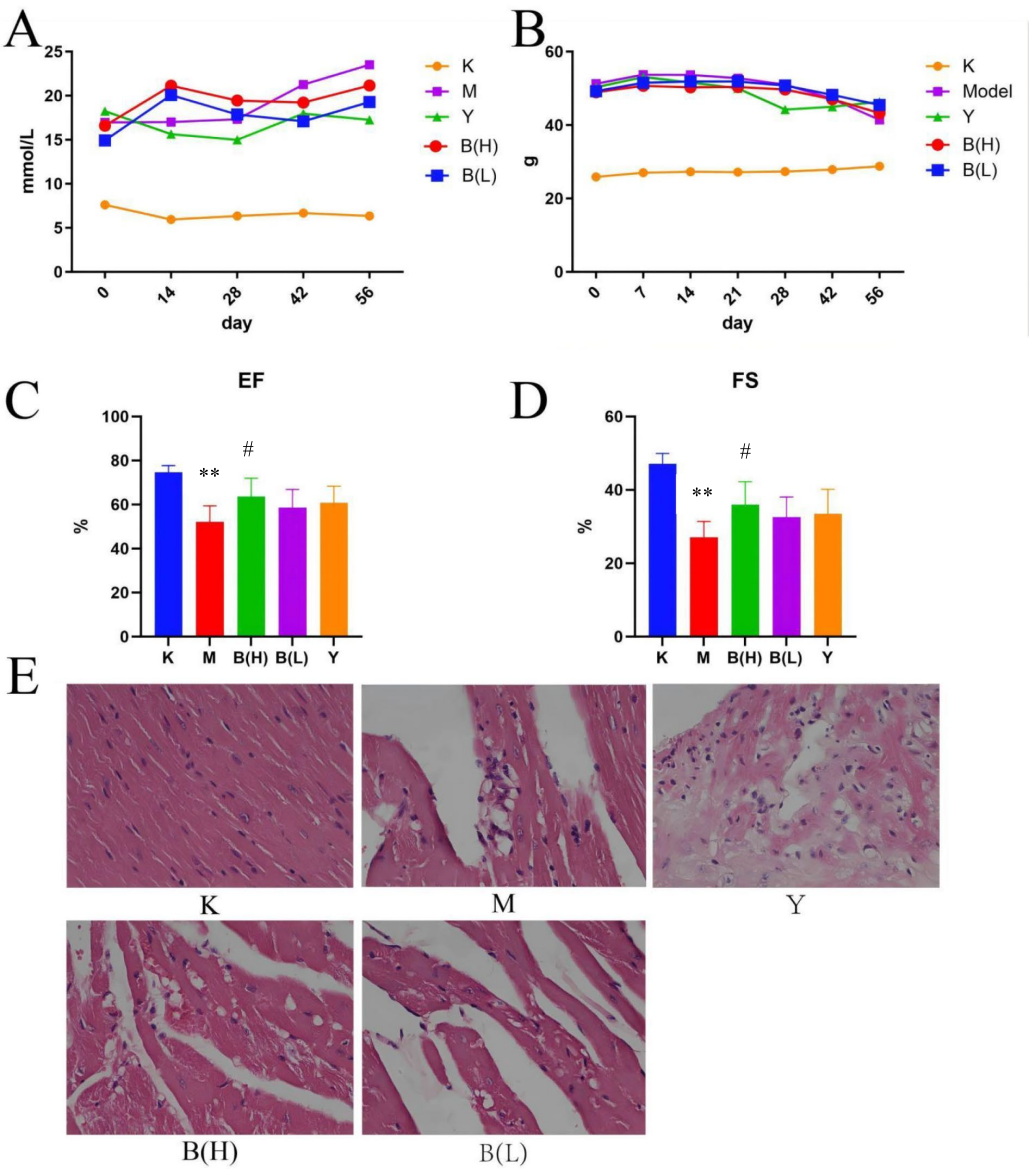
The effect of SP on glucose metabolism and protect cardiac function in db mice. **(A)** Changes in mouse body weight among each group. **(B)** Changes in blood sugar of mice among each group. **(C)** Ejection fraction (EF%). **(D)** shortening fraction (FS%) Compared with the control group. **(E)** The pathological changes of heart in control group(×20). (K-control group; M-model group; Y-active control group; B(H)-high-dose SP group; B(L)-low-dose SP group) compared with the control group, ^* *^p < 0.01; Compared with model group, ^#^*p* < 0.05.

The results of the repeated measurements variance analysis on the body weight of each group of mice indicated a significant difference between the control group and the model group (*P*<0.05). In terms of blood glucose levels, a particularly significant difference was noted between the blank group and the model group (*P* < 0.05). These results suggest that Huangqin decoction and the high- and low-dose groups of SP had no significant impact on the body weight and blood sugar of diabetic mice. Detailed findings are presented in Figures 1A,B.

**Figure 2:**
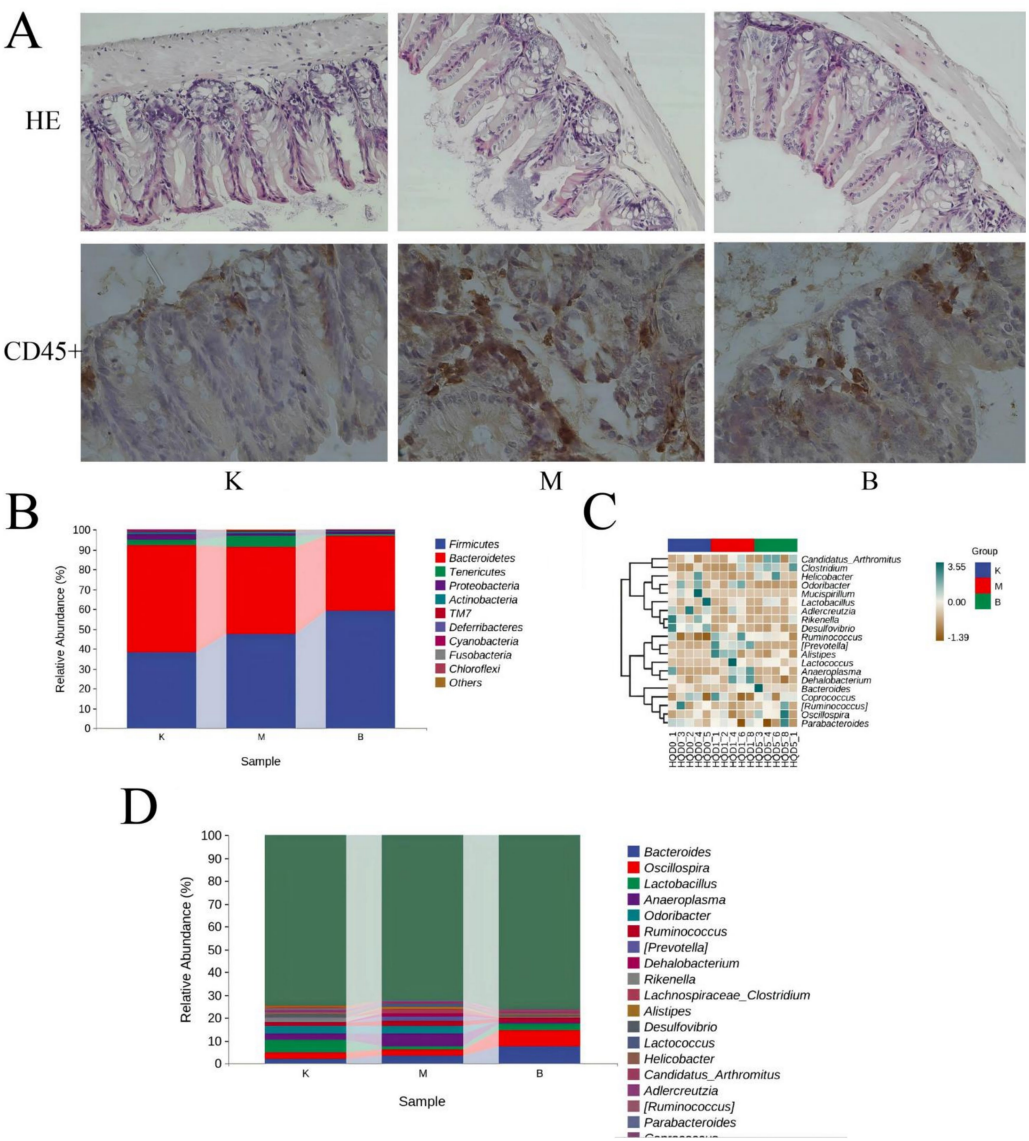
Influence of intestinal mucosal barrier integrity and the intestinal flora in T2DM model mice. **(A)** Histomorphological (×20) and immunohistochemistry of CD45 + (×40) changes in the cecum of db mice. **(B)** Distribution of dominant bacterial species in mouse gut microbiota. **(C)** Thermal analysis plot of community taxonomic abundance distribution of the top 20 absolutely dominant mouse bacterial genera. **(D)** Distribution of the top 20 dominant genera in the mouse flora.

H&E staining revealed that the nucleus of myocardial cells in the control group was normal and the cytoplasm was pink. In the model group, obvious myocardial cell necrosis was observed, necrotic myocardial cells were constricted and dissolved, and myocardial stripes disappeared. In the positive drug group, obvious myocardial cell necrosis, inflammatory cell infiltration, collagen fiber proliferation, and myocardial stripes disappeared; SP showed sporadic myocardial cell necrosis in the high dose group, and myocardial stripes disappeared slightly; SP showed sporadic myocardial cell necrosis, inflammatory cell infiltration, collagen fiber proliferation, and slight disappearance of myocardial (Figure 2E). After the mice in each group were anesthetized by intraperitoneal injection of sodium pentobarbital, the cardiac function of the mice in each group was measured by echocardiography, and the left ventricular ejection fraction (EF%) and fractional shortening (FS%) were measured. Ultrasound results showed that compared with the control group, the EF% and FS% values of the mice in the model group were significantly decreased (*p* < 0.01); compared with the model group, the EF% and FS% values of the high-dose group of SP were significantly increased (*p* < 0.01, *p* < 0.05), the EF% and FS% values of the other groups were not significantly different from the model group (*p* > 0.05), as shown in Figures 1C,D.

### 3.2 SP has a protective effect on the intestinal mucosa of diabetic rats

**Figure 3:**
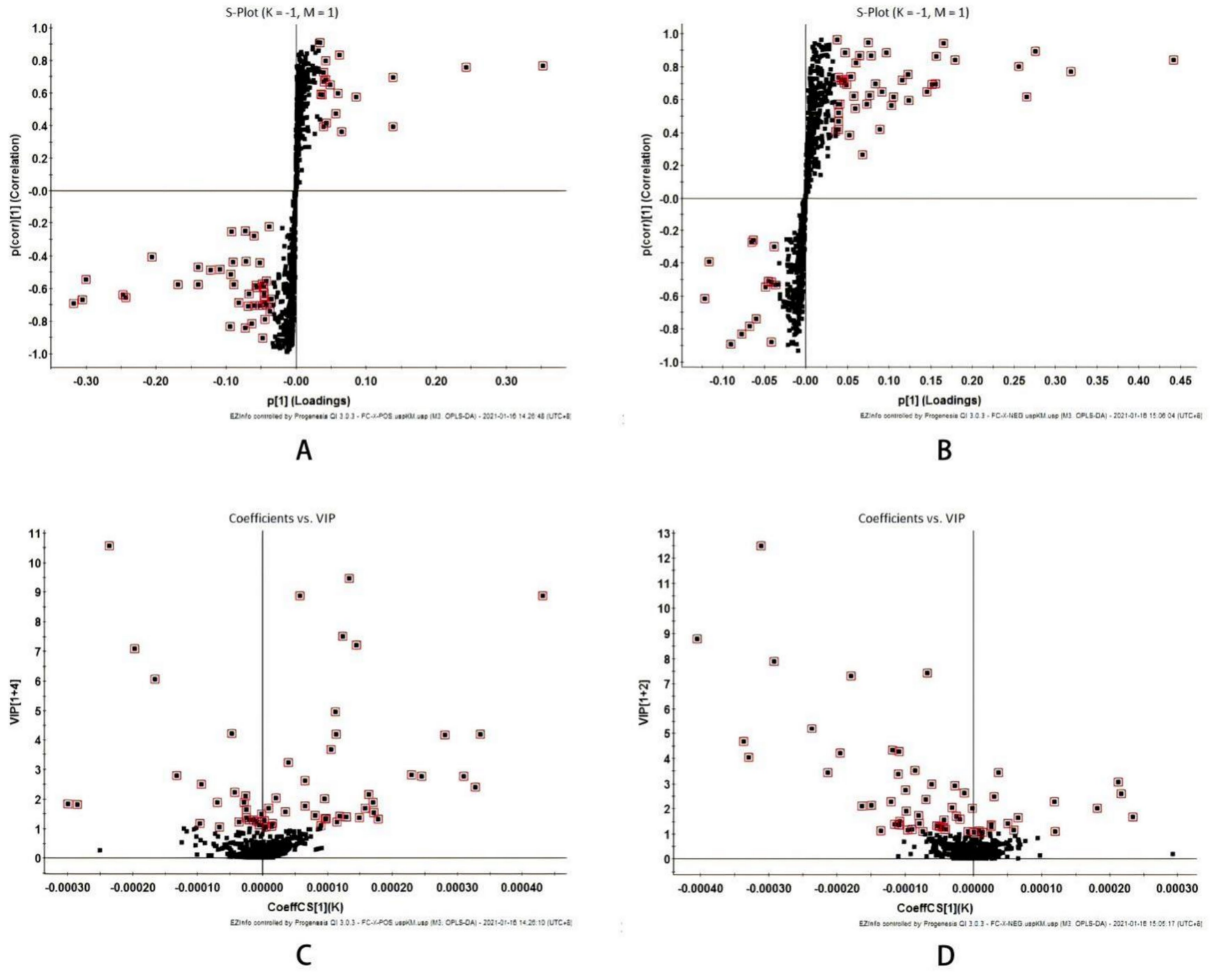
Multivariate statistical analysis of liver metabolism spectrum in intestinal mucosal damage of diabetes mice **(A)** S-plot plots of positive ion mode and **(B)** Negative ion mode; **(C)** VIP plot of positive ion mode and **(D)** Negative ion mode; VIP > 1.

To some extent, the degree of damage to intestinal barrier integrity reflects the barrier function of intestinal mucosa. H&E staining was used in this work to assess the change in intestinal barrier function by measuring the length of intestinal villi and the thickness of the muscular layer. H&E staining revealed that the villus length and muscular layer thickness of the intestinal mucosa were normal in the control group; in the model group, the villi were clearly reduced and the muscular layer thickness was decreased. However, as compared to the model group, the symptoms of SP in each dosage group and the positive control group were improved, and the villi length was similar to that of the control group, but the muscular layer thickness was still much lower. The group with the highest dosage has the most significant effect (see Figure 2A). The intestinal mucosa was sectioned, and variations in leukocyte infiltration were observed using CD45 staining, as shown in Figure 3. Only a few CD45+ cells infiltrated the control group. The model group had significantly higher infiltration of CD45+ cells than the control group, but the SP medicine treatment groups and the positive control group had significantly lower infiltration than the model group (Figure 2B).

As shown in Figure 2C, from the phylum level, *Firmicutes* and *Bacteroidetes* are the most important phylum, followed by *Tenericutes*, *Proteobacteria*, and *Actinobacteria*. These five phylum account for a very high proportion of the whole phylum, and belong to absolute dominant phylum. Compared with the control group, in the model group, *Firmicutes* (38.06% → 47.37%), *Bacteroidetes* (54.25% → 43.77%), *Tenericutes* (2.48% → 5.55%), *Proteobacteria* (2.98% → 1.46%), *Actinobacteria* (0.89% → 0.73%), *Firmicutes / Bacteroidetes* ratio in the blank group was 0.70, and *Firmicutes / Bacteroidetes* ratio in the model group was 1.08, suggesting that the dominant flora of model group mice and blank group mice had changed significantly in structure; Compared with the model group, the ratio of *Firmicutes / Bacteroidetes* of SP group was 1.57, suggesting that SP group could regulate the dominant flora of db / db mice.

From the genus level, through cluster analysis of the absolute dominant genera in the top 20, the relative abundance of different genera was found and analyzed. They are *Bacteroides*, *Oscillospira*, *Lactobacillus*, *Anaeroplasma*, *Odoribacter*, *Ruminococcus*, *Lactococcus* etc., Figures 2D,E.

### 3.3 Effects of SP on metabolites and metabolic pathways in intestinal tissues of diabetic rats

#### 3.3.1 Analysis of mice serum metabolism

**Figure 4:**
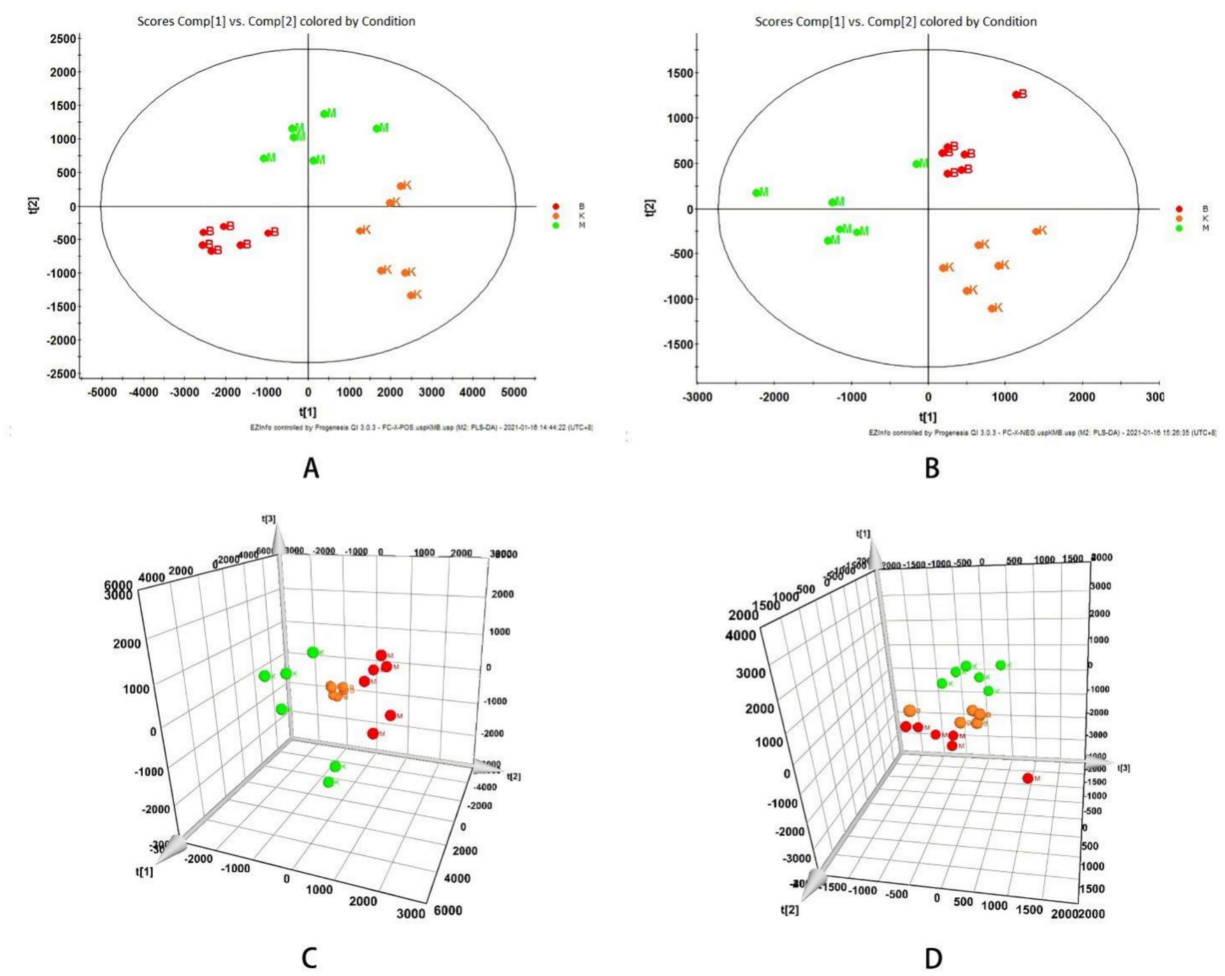
Influence of intestinal mucosal barrier integrity and the intestinal flora in T2DM model mice. **(A)** Histomorphological (×20) and immunohistochemistry of CD45 + (×40) changes in the cecum of db mice. **(B)** Distribution of dominant bacterial species in mouse gut microbiota. **(C)** Thermal analysis plot of community taxonomic abundance distribution of the top 20 absolutely dominant mouse bacterial genera. **(D)** Distribution of the top 20 dominant genera in the mouse flora.

The data obtained by UPLC-TOF-MS was analyzed, and PCA was processed using unsupervised orthogonal partial least squares discriminant analysis to illustrate the metabolic profile of mice serum samples. The results show that the control group is clearly separated from the model group, indicating that the model produced in this research has a high reliability. When the control, model, and SP groups are compared at the same time, it is found that each group has its own clustering, and the SP group has a tendency to return to the control group, indicating that SP group can improve the metabolism of mice in model group, as shown in Figure 4.

The S-plot diagram shows that the majority of metabolite ions cluster near the origin, with just a few ions deviating from the origin. These ions deviate from the dot, indicating that the two groups are different. By adopting the screening condition VIP > 1, the VIP-plot diagram can be obtained and possible biomarkers can be pre-selected, as illustrated in Figure 3.

#### 3.3.2 Endogenous potential biomarkers of serum metabolism in mice

**Figure 5:**
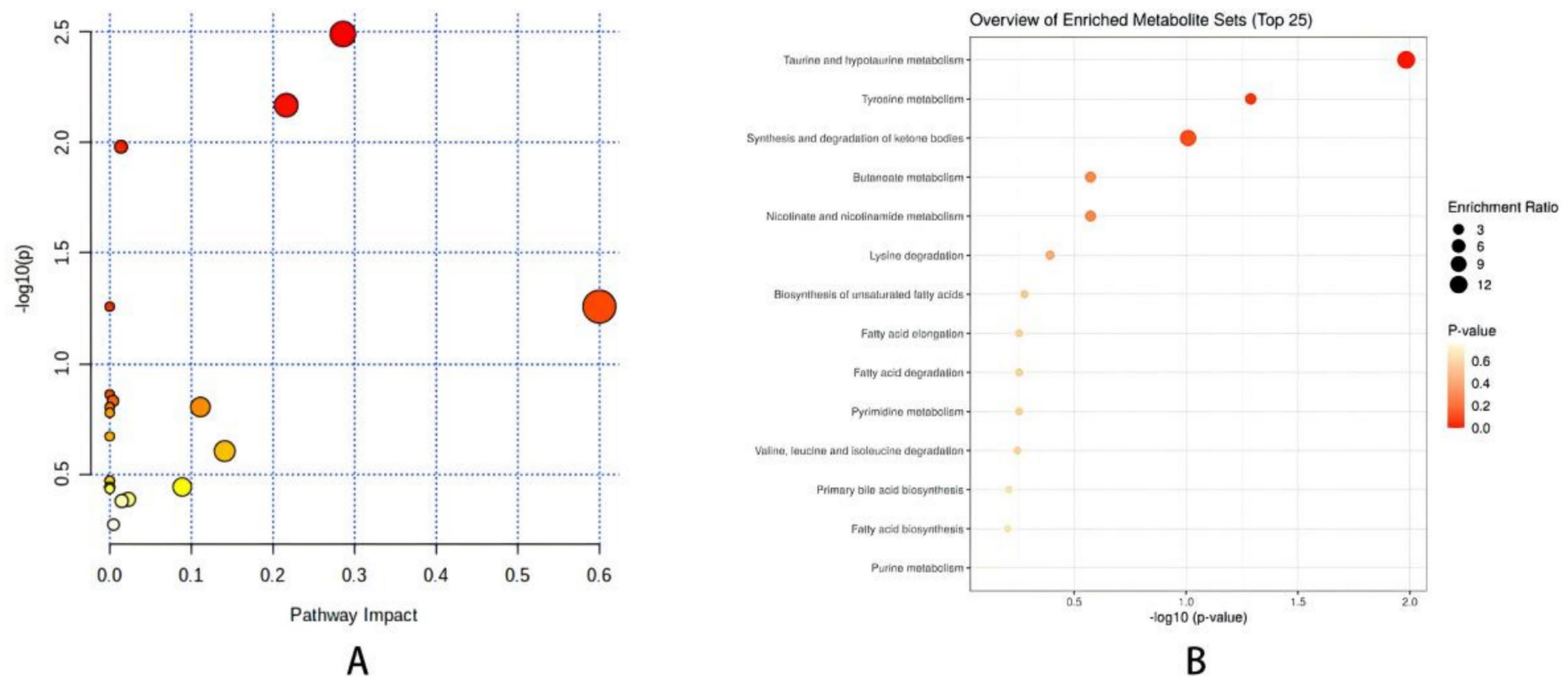
**(A)** Metabolite enrichment analysis bubble diagram. **(B)** Metabolic pathway analysis <0.01, *p < 0.05. Comparison with model group ^##^*p* < 0.01, ^#^*p* < 0.05.

Through the prediction of the changes of serum metabolites in mice, find the metabolites in line with VIP > 1, fold change >1.2 or < 0.8 and Q-value >0.5. Compare the blank group with the model group and find 52 differential metabolites. Compare the model group with the SP group and find 49 differential metabolites. The intersection of the above comparison can get 31 common differential metabolites; After the comparison of ionic strength, it was found after the administration intervention that the SP group had a callback on 21 indexes, which could significantly up regulate 9 biomarkers (*p* < 0.01), significantly up regulate 2 biomarkers (*p* < 0.05), significantly down regulate 5 biomarkers (*p* < 0.01), and significantly down regulate 5 biomarkers (*p* < 0.05), as shown in Figure 5 and Table 1.

## 4 Discussion

The gut microbiome and its byproducts play an essential role in metabolic syndrome and cardiovascular well-being, influencing conditions such as obesity, diabetes, atherosclerosis, hypertension, and heart failure. As a result, there is an urgent demand for safe medications with minimal adverse effects to manage disorders related to intestinal microbiota and metabolites in diabetic individuals. This study reveals that diabetes inflicts cardiac harm in mice, which is associated with imbalances in gut microbiota and metabolites. Combinations of Traditional Chinese medicine, such as SP, which are frequently utilized to manage enteritis, demonstrate regulatory and protective actions against damage to the intestinal barrier, as well as imbalances in gut microbiota and metabolites, according to experimental results. These discoveries provide new insights into therapeutic strategies for diabetic cardiomyopathy that prioritize the rectification of disturbances in gut microbiota and metabolites.

Recent research has revealed that both *Scutellariae Radix* and *Paeoniae Radix Alba* root provide a protective effect on the intestinal tract, as demonstrated in various animal studies and clinical trials. Our investigation shows that these medicinal components reduce intestinal inflammatory responses by influencing the metabolism of intestinal *Helicobacterium*, which includes substances such as Palmitic acid and bile acids, and also affect the metabolic processes in diabetic heart tissue. By employing metabolomics, we pinpointed five significant metabolites (PC(18:0/18:2(9Z,12Z)), LysoPC(15:0), LysoPC(20:1(11Z)), PE(18:2(9Z,12Z)/18:1(9Z)), Glycerophosphocholine) along with two related metabolic pathways (Arachidonic acid metabolism, Glycerophospholipid metabolism). These results indicate a protective role for the hearts of diabetic rats.

The results showed that the abundance of *Helicobacter* in diabetic intestinal mucosal injury model mice increased significantly. *Helicobacterium* is considered to be an intestinal pathogen, belonging to *Helicobacter*. As *Proteobacterium*, its outer membrane is mainly composed of lipopolysaccharide, so its abundance increases to produce a large amount of LPS, which causes the body’s oxidative stress to increase, resulting in a large consumption of PUFAs-containing lipids (Sevanian and Hochstein, 1985). In this study, Palmitic acid showed significant differences among the groups, Palmitic acid exhibits a positive correlation with Helicobacterium, which can enhance the production of palmitic acid and contribute to lipid metabolism disorders. Furthermore, palmitic acid is capable of inducing apoptosis in islet *β* cells, a process associated with free fatty acid (FFA) activation of endoplasmic reticulum stress (ERS) and the downregulation of the anti-apoptotic factor Bcl-2. This suggests that palmitic acid may diminish insulin synthesis and secretion by inducing apoptosis in β cells (Litwak et al., 2015). Some scholars stimulated rat islet β cells with palmitic acid, and found that the expression of Ca^2+^- adenosine triphosphate (ATP) enzyme phenotypes 1 and 4 in rat islet β cell membrane was inhibited, Ca^2+^ influx was obviously insufficient, and cell excitability was decreased(Acosta-Montaño et al., 2019). Liu et al. (2011) found that palmitic acid decreased insulin signal transmission by regulating NF-κB pathway. Alipourfard et al. (2019) also found that lipid metabolism disorder can weaken insulin signal transmission through TNF-*α* pathway. Mukhuty et al. (2021) found that palmitic acid can up-regulate the expression of inflammatory immune factors such as IL-1β and IL-6 by activating TLR4-NF-κB pathway, thus down-regulating the Akt phosphorylation level and the expression of glucose transporter 2 protein in mouse islet cells, and inhibiting the transmission of insulin signal, thus causing lipid metabolism disorder. In this study, palmitic acid was enriched into three pathways: Fatty acid degradation, Fatty acid biosynthesis and unsaturated fatty acid biosynthesis. Compared with the control group, the level of palmitic acid in the model group was significantly higher, while SP had a callback effect on the above-mentioned different metabolites. In conclusion, SP may control palmitic acid, which in turn can regulate the metabolic pathway disorders of fatty acid degradation, fatty acid biosynthesis, and unsaturated fatty acid biosynthesis, therefore preventing and treating disease.

Pathway enrichment analysis of the differential metabolites in this study indicates that SP can regulate taurine and hypotaurine Metabolism. Free bile acids combine with glycine and taurine to form combined bile acids, and almost all bile acids in mice combine with taurine. Studies show that bile acids can regulate lipid metabolism and sugar metabolism by activating FXR or TGR5, which explains why abnormal primary bile acids can lead to lipid metabolism disorder (Wang J. et al., 2017). The primary bile acids can be converted into secondary bile acids by the actions of uncoupling, oxidation and epimerization, 7- dehydroxylation, esterification and desulfurization of bile acids. Primary bile acids increase intestinal permeability through autophosphorylation, dephosphorylation and tight junction rearrangement of epithelial growth factor receptors, which leads to endotoxemia, IR and inflammatory cytokine release (Miele et al., 2009; Raimondi et al., 2008; Forlano et al., 2022). Bile acids interact with intestinal flora in a two-way. Under the high-fat diet rich in SFAs, intestinal flora is out of balance, which leads to the increase of bile acid reabsorption and changes of its components, and finally the significant increase of bile acids secreted and excreted. In the intestinal tract, a large number of intestinal flora compete for limited energy sources, and bile acids have strong antibacterial ability, especially DCA, which changes the pH value in the intestinal tract and exerts strong selective pressure on the intestinal flora. However, the slight difference in sensitivity to DCA is obviously amplified, which leads to a drastic imbalance of the flora. Only the species with strong adaptability can maintain and expand their own flora advantages. Because *Bacteroides* is more sensitive to DCA, Moreover, *Actinomycetes* stimulate bile acid synthesis, while *Bacteroides* and *Proteobacteria* inhibit bile acid synthesis, while *Bacteroides* is related to bile acid metabolism, and it can promote the decomposition of taurine combined with bile acid in serum. Taurine is a derivative of cysteine, which is called conditionally essential amino acid. Studies suggest that it has therapeutic effect on diabetes-related complications, and its mechanism may be related to membrane stability, anti-inflammatory and antioxidant stress(Das et al., 2012).

In this investigation, taurine concentration was enhanced within the taurine and hypotaurine metabolic pathway. When compared to the control group, the model group exhibited a markedly reduced level of taurine, while SP demonstrated a restorative effect on taurine levels. Consequently, we hypothesize that SP may influence taurine content by impacting *Actinomycetes, Bacteroides, and Proteobacteria*, thereby modulating the imbalance in the taurine and hypotaurine metabolism pathway, which could contribute to the prevention and management of related diseases.

## 5 Conclusion

This research reveals that mice with diabetic cardiomyopathy experience compromised intestinal microbial balance and barrier function, resulting in cardiac dysfunction. Additionally, treatment with SP alleviates damage to the intestinal barrier and maintains the intestinal microbiome, which in turn enhances the disorders related to glucose and lipid metabolism. Notably, alterations in serum metabolites due to diabetes affect the metabolites found in myocardial tissue, ultimately providing protective benefits to the heart. In conclusion, our findings indicate that SP reduces intestinal microbial damage and barriers’ dysfunction, enhances glucose and lipid metabolism, and lessens cardiac injury. Overall, these results underscore the importance of gut microbiota along with glucose and lipid metabolism in cardiac damage stemming from diabetes, offering critical insights for developing potential therapeutic approaches.

## Data Availability

The raw data supporting the conclusions of this article will be made available by the authors, without undue reservation.
